# Cinematographic Recording of a Metastable Floating Island in Two- and Three-Dimensional Crystal Growth

**DOI:** 10.1021/acscentsci.2c01093

**Published:** 2022-12-20

**Authors:** Masaya Sakakibara, Hiroki Nada, Takayuki Nakamuro, Eiichi Nakamura

**Affiliations:** † Department of Chemistry, The University of Tokyo, 7-3-1 Hongo, Bunkyo-ku, Tokyo 113-0033, Japan; ‡ Environmental Management Research Institute, National Institute of Advanced Industrial Science and Technology (AIST), 16-1 Onogawa, Tsukuba 305-8569, Japan

## Abstract

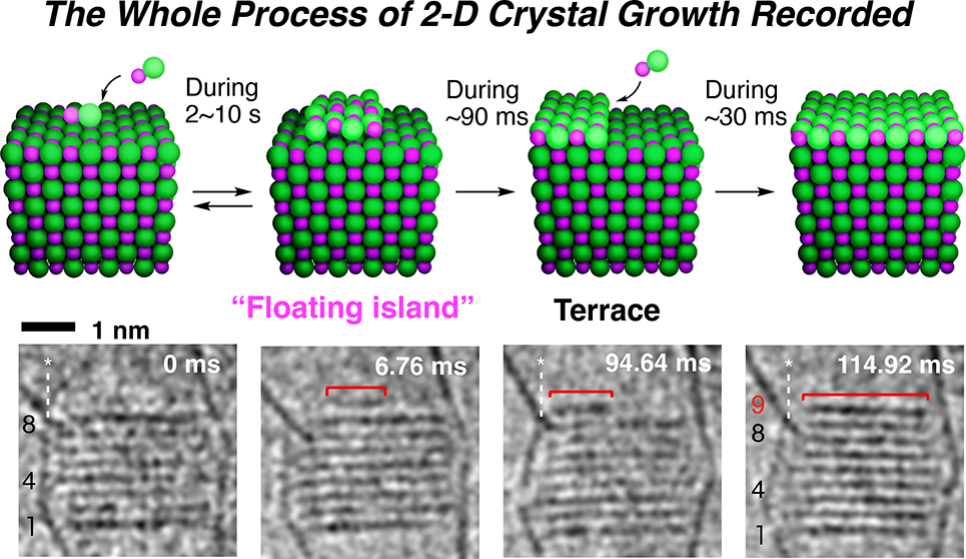

Many chemical reactions go through a cascade of events in which a series of metastable intermediates appear, and crystal nucleation is no exception. Although the consensus on the energetics of nucleation suggests the formation of metastable states preceding the crystal growth, little experimental evidence has been reported for their dynamics at an atomistic level. Operando imaging of two-dimensional nucleation on a defect-free NaCl nanocrystal in carbon nanotubes using a millisecond angstrom-resolution transmission electron microscope revealed the formation of a metastable “floating island” (FI) that migrates thermally on the (100) facet of NaCl as the first intermediate of epitaxy. The speed of the migration at 298 K is estimated to be larger than 0.3 nm ms^–1^. When a crystal tumbles in a container, a space repeatedly forms between the crystal and the container wall that hosts the FI. Tumbling changes the surface energy repeatedly and promotes the conversion of the FI into a new epitaxial layer. We anticipate that this surface catalysis mechanism found on the nanoscale also operates in bulk heterogeneous nucleation where agitation and attrition accelerate crystallization.

## Abstract 2

Atomic resolution cinematographic imaging of two-dimensional epitaxy on a defect-free NaCl nanocrystal revealed the formation of a transient mobile cluster at the very beginning of the epitaxy.

## Introduction

**Figure 1:**
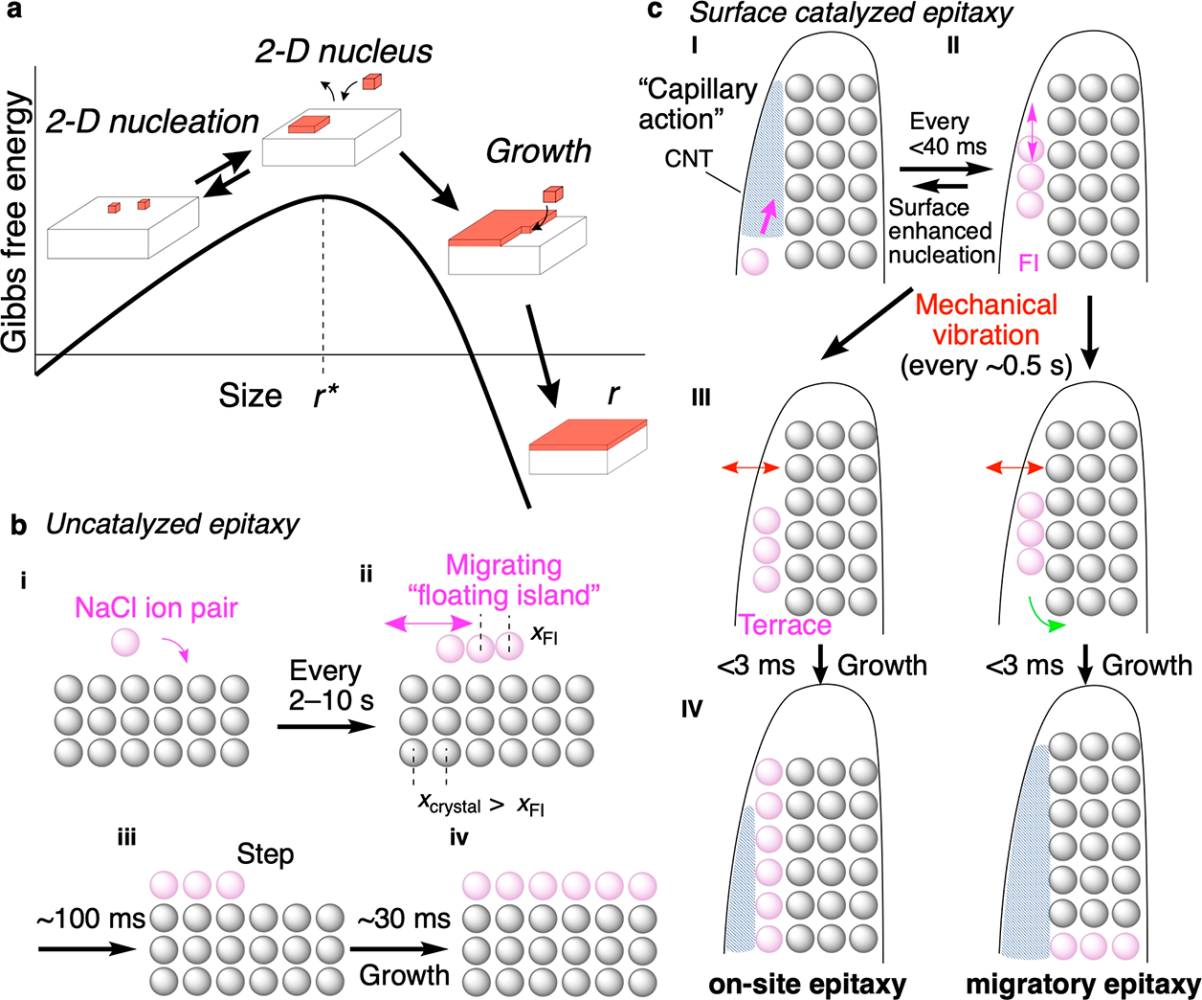
Schematic diagrams of crystallization. (a) Energy diagram of 2-D nucleation and growth. The size of a 2-D critical nucleus is shown as r*. (b) 2-D epitaxy on a defect-free facet. (c) Nucleation and growth by surface catalysis. The NaCl ion pairs were supplied from a hidden reservoir located at the bottom of the CNT. Red arrows illustrate the vibration of CNT. Migration is shown with a green arrow. Blue mesh presents a low potential space that attracts ion pairs by capillary action. The ion pairs are supplied from the bottom of CNT and form an FI and epilayer on the crystal surface.

Nucleation constitutes the least-understood stage in the production of a new phase in such diverse areas as cloud formation,^1^ self-organization phenomena found in medication and pathology,^2−4^ and crystallization processes central to laboratory experiments and industrial processes.^5^ The current consensus on the energetics of nucleation considers metastable states along an uphill path to a critical crystal nucleus (Figure 1a).^6,7^ A cascade of intermediates emerge stochastically in the metastable states to finally generate a transient crystal nucleus that grows irreversibly into a crystal.^8−10^ However, little has been reported on the dynamics of the metastable states at an atomistic level. The events often occur too rapidly, beyond the spatiotemporal capability of available analytical methods,^11,12^ including in situ transmission electron microscopy (TEM) and scanning probe microscopy whose resolution seldom reaches angstrom and millisecond (ms).^13^ Having recently developed an experimental setup where a NaCl nanocrystal (NC) grows spontaneously in a closed carbon nanotube (CNT) containing a supply of NaCl ion pairs,^14^ we performed operando imaging of two-dimensional (2-D) nucleation on a defect-free (100) surface of the NC using single-molecule atomic-resolution time-resolved electron microscopy (SMART-EM).^14−17^ Here we can study the crystal growth from the side orthogonal to the direction of epitaxy.^18,19^ We describe here the formation and dynamics of a metastable “floating island” (FI) as the first intermediate of two-dimensional epitaxy that has shorter interionic distances than those in the crystal (cf. Figure 1b, where *x*_FI_ is shorter than *x*_crystal_). While the FI formation occurs stochastically and infrequently (once during 2 to 10 s), the FI formation and the epitaxy occur more frequently by 1 order of magnitude when the NC tumbles in the CNT (Figure 1c).^20^ It is because the FI formation occurs frequently in a nanospace between the NC and the CNT wall due to capillary action (I to II); that is, like any nano space, this space has a low potential (blue mesh) and attracts ion pairs to form a FI. The FI either forms a new epitaxial layer on the same facet (on-site epitaxy, III to IV) or on a different facet when the FI is squeezed out from the nanospace by CNT vibration (migratory epitaxy). One cycle of surface-catalyzed epitaxy thus ends. Since 3-D crystal growth comprises a series of 2-D nucleation/growth events, the mechanism described here will be pertinent to a wide variety of 3-D crystal growth processes. The present work also provides a further demonstration of the value of the cinematographic study of stochastic chemical events to access hitherto unavailable information on the interactions of molecules and ion pairs^21,22^ such as molecular shuttling,^16^ C–C bond-forming reactions,^23^ and 3-D crystal nucleation recorded with millisecond angstrom resolution.^14^

## Results and Discussion

**Figure 2:**
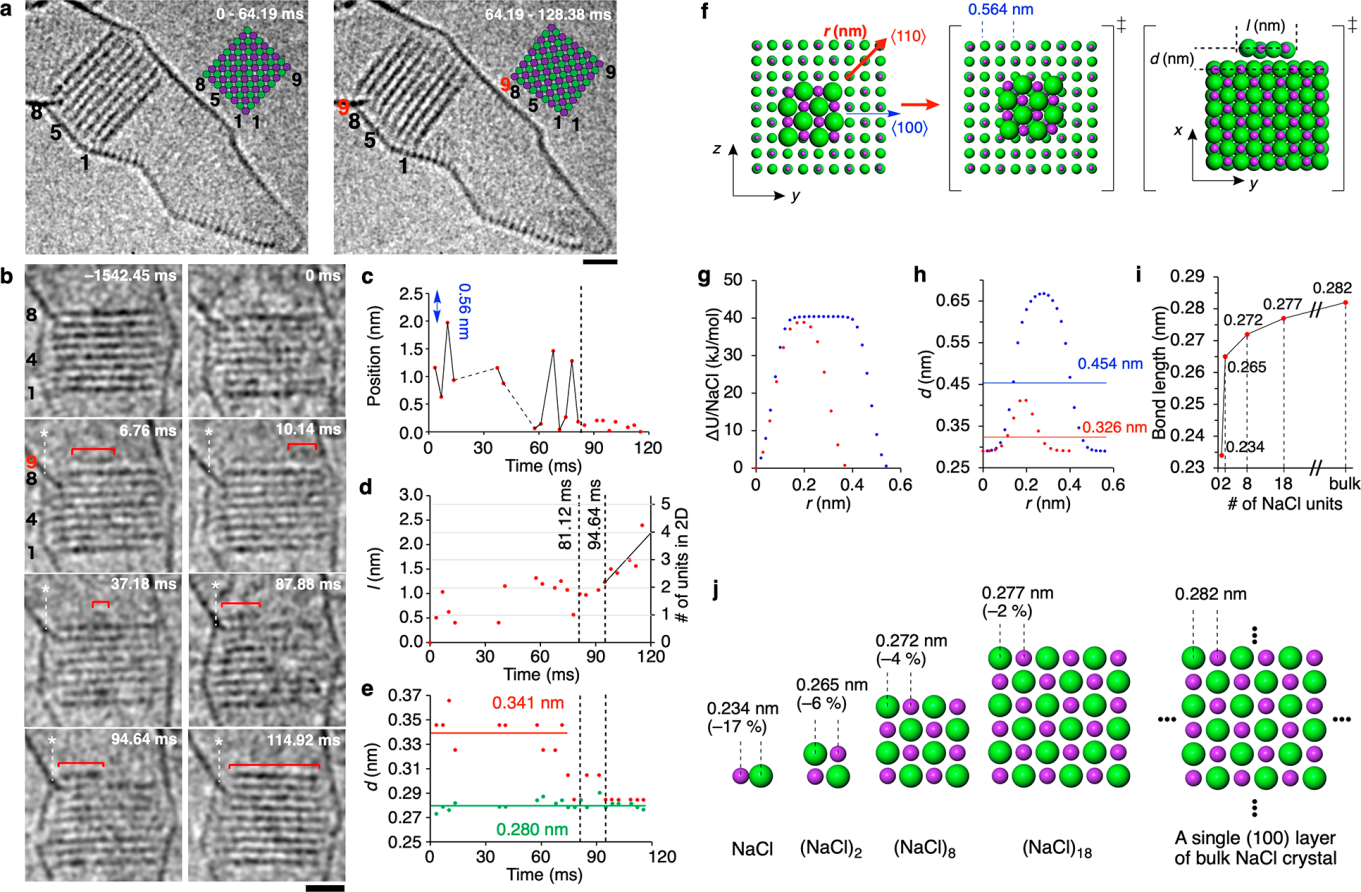
Observation of migrating FI and lateral growth of terrace using a K3-IS camera. (a) 20-frame stacked images of growth of an (8,9) NC to a (9,9) NC. (b) Representative TEM images of NaCl lateral growth (see Videos S1–S2, Figure S3). Surface clusters are indicated with red bars. Scale bar: 1 nm. (c) Time evolution of the position of the surface cluster. Position is defined as the distance between the left wall of the CNT and the left edge of the cluster. (d) Time evolution of the length of the surface cluster. The black solid line indicates the zeroth-order growth of the terrace size. (e) Time evolution of interlayer distances on the surface cluster (red) and average value on lattice planes (green). Data distribution is due to the 0.020 nm pixel size. (f) Monte Carlo simulation of the trajectory of surface cluster migration without CNT using a Coulomb potential and Lennard–Jones potential (see Supporting Information for details).^27^ (g) Potential curves of FI migration in two trajectories. (h) Evolution of *d* in the FI migration. Solid lines indicate the time-averaged *d* value. (i) Na–Cl distance in square NaCl clusters differing in size in a vacuum. (j) Single-layered NaCl clusters exhibit large size-dependency of the Na–Cl distance, cf. Figure 2i. The NaCl crystal data show only one layer.

We first discuss the uncatalyzed on-site epitaxy relevant to the scheme in Figure 1b. As previously reported, the NaCl@CNT specimen was prepared by first immersing water miscible aminated CNT^24^ in aqueous NaCl followed by drying in a vacuum.^14^Figure 2a shows two consecutive 64.19 ms frames, illustrating how a new layer (#9) forms on the (100) plane of an 8 × 9 array of NC (denoted as (8,9) NC) to form a (9,9) NC immobilized in the tight-fitting interior of a CNT under 1 × 10^–5^ Pa. Layer #8 is flat and defect-free (see Figure 1b top). We surmise that this NC is 9 × 9 × 8 in size (648 atoms) or 9 × 9 × 10 (810 atoms) considering charge neutrality and the conical shape of the CNT. In a single-shot 1.69 ms raw image, the crystal was hardly visible but was made visible after low-pass filtering and 4 × 4 × 2 pixel binning (Figure S1). Gaussian filtering further improved the image quality with minimum loss of time resolution, a 3.38 ms frame^–1^ speed, and pixel resolution of 0.020 nm.

Cinematographic and theoretical data in Figure 2b–d illuminate the details of the pathway of uncatalyzed epitaxy probed at a previously unachievable level. Figure 2b shows eight frames from a video taken for a total duration of 2 s with a frame rate of 3.38 ms frame^–1^. They show the time evolution of the formation and thermal migration of an FI (Figure 2c) and the epitaxy of layer #9 on #8 over 1.7 s (−1542.45 to 114.92 ms). We set the frame time 0 as the frame just before the appearance of the FI. The FI formation event, and hence the crystal growth event on the defect-free (100) face, were entirely stochastic and rare, with a frequency of once in 2 to >10 s, as analyzed for several NCs. This observation lends experimental support at an atomistic level that nucleation on a defect-free crystal plane is an unfavorable process, as has often been mentioned in the literature (cf. i and ii, Figure 1b).^25,26^

In the 3.38 ms frame, an FI that formed initially was 0.5 nm in length (*l*), equivalent to one NaCl unit (Figure 2d). It migrated stochastically, changing its size between one and two units and disappearing twice, probably due to swift motions (Figure 2c). Mapping the translation along the time, Figure 2c allows us to estimate the minimum speed for migration at 298 K to be ∼0.3 nm ms^–1^, 0.3 μm s^–1^, or 1.1 mm h^–1^. The most characteristic parameter is the large value of the interlayer distance, *d*, which averages 0.34 nm (Figure 2e). This value of *d* is 23% longer than the one found in bulk crystal (0.282 nm at 298 K, Figure S2), indicating that the FI is floating over the atomistically rough (100) facet. At 81.12 ms, the FI touched the left wall of the CNT, lost its kinetic energy, and landed on layer #8 (note a sudden decrease of *d* to 0.28 nm, Figure 2e). This FI forms as the last metastable species before irreversible crystal growth and hence can be regarded as a critical crystal nucleus of 2-D epitaxy ((NaCl)_8_, Figure 1a). This FI is not any more floating and hence must regarded now as a “terrace,” which started to grow rapidly into a new layer #9 at 94.92 ms and was completed in ∼30 ms (Figure 2d,e).

Careful inspection of Figure 2d shows a few remarkable pieces of atomistic information on the FI formation and the step growth (Figure 1c). First, the size of the FI fluctuated between one and two NaCl units during 1.9 s as we monitored the whole process of epitaxy for 2 s (Figure 2a shows only the end of the TEM observation). This indicates that FI represents the metastable state before r* in Figure 1a. Second, once the FI formed an immobile terrace at 81.12 ms, the terrace grew larger quickly by 0.5 NaCl unit with a zeroth order rate after 94.64 ms, that is, epitaxy by one single NaCl row after another. Assuming that the NC size is 9 × 9 × 8 (taking a round cross-section of CNT), we calculated the rate constant of the growth to be roughly 900 NaCl s^–1^. Such a higher frequency of the step growth than the FI growth (i.e., little growth) is not unexpected because a migrating ion pair should combine with a terrace far more easily than with another migrating ion pair.^28^

We can consider a priori two trajectories of the FI migration on the (100) facet, <100> (blue) and <110> (red) directions. We found that the <110> path is favored according to a Monte Carlo simulation of the trajectory of a 16-atomic square NaCl cluster on a 648-atomic crystal at 298 K (Figure 2f). Figure 2g,h compares the two trajectories for their energetics and the distance *d* against the displacement (*r*) from the original location. The <110> trajectory has a narrower kinetic barrier and a smaller maximum *d* value, requires less work, and should be preferred over the <100> trajectory. Given that the FI migrates with a minimum speed of 0.3 nm ms^–1^ and the imaging frame rate is 3.38 ms frame^–1^, the experimental distance *d* = 0.341 nm must be time-averaged and expectedly matches well with the theoretical average of *d* = 0.326 nm for the <110> trajectory (instead of 0.454 nm for <100>). The migration trajectory in Figure 2c provides other supporting data, as it shows that the FI migrates often with a step shorter than 0.56 nm, which is incompatible with the <100> trajectory (cf. blue arrow in Figure 2f).

Why does the FI “floats” over the (100) facet with *d* = 0.341 nm? It is because of a large mismatch of the lattice size between a small single-layer NaCl and a NaCl crystal, as illustrated in Figure 1b, where *x*_FI_ is shorter than *x*_crystal_, and detailed in Figure 2i,j. Figure 2j on the left shows a monomeric NaCl ion pair with a 0.234 nm atomic distance, 17% shorter than the 0.282 nm distance found in the crystal (on the right). As the cluster size becomes larger, the nearest ionic interaction weakens, and the distance increases to 0.272 nm (4% shorter) for (NaCl)_8_ and 0.277 nm (2% shorter) for (NaCl)_18_ (i.e., 6 × 6 cluster). Given that a lattice mismatch smaller than 5% is favored for epitaxy,^29−33^ we consider that the (NaCl)_8_ cluster could “land” only when it lost the kinetic energy at 81.12 ms by touching the left CNT wall (Figure 2c).

**Figure 3:**
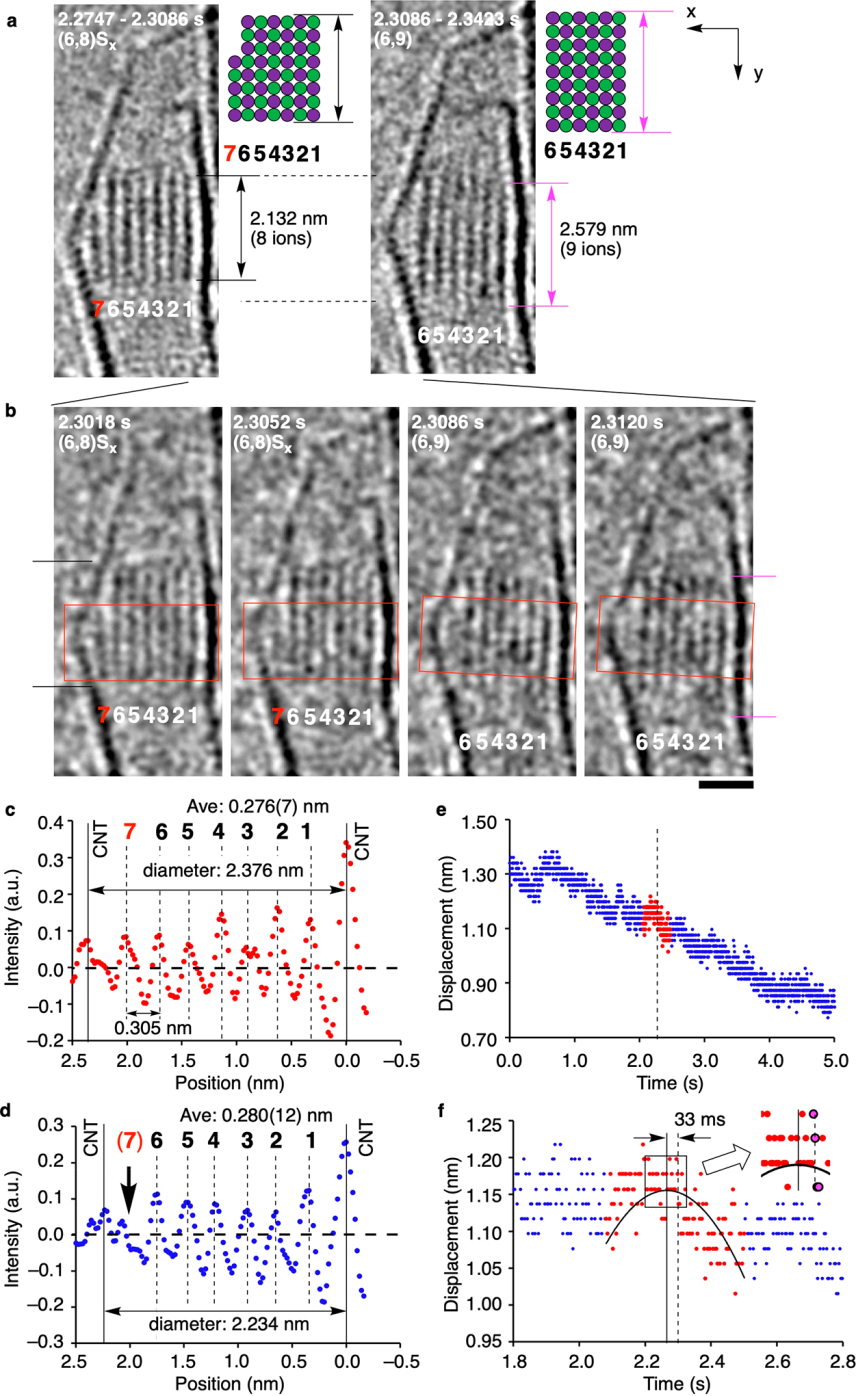
Observation of migratory epitaxy. (a) 10-frame stacked TEM images of migratory epitaxial growth of a NaCl NC. (b) Single-shot TEM images with a frame rate of 3.38 ms frame^–1^ (see Video S3). EDR = 2.2 × 10^6^ e^–^ nm^–2^ s^–1^, after image processing and Gaussian filtering (Figure S4). Scale bar: 1 nm. (c, d) Interlayer distance measurement at 2.3018 and 2.3120 s, respectively. Intensity profiles were taken from red squares in panel b. See Figure S5 for profiles taken from four sequential frames. (e) A vibrational plot of the CNT. The persistent decrease of the displacement value is due to the thermal drift of the specimen. The dashed line indicates the frame of crystal growth. (f) Correlation between vibration (quadratic simulation of red dots in black, and FI migration (dashed line). The FI migration occurred at 2.3086 s (dashed line), 33 ms after the CNT vibration was at its maximum amplitude at 2.2750 s (solid line). Inset: purple dots refer to four images in panel b. From the quadratic curve, we estimate the acceleration felt by NaCl@CNT to be 4.4 femtometer ms^–2^.

Figure 3a shows an example of migratory epitaxy (Figure 1c) via an FI trapped in a space between an NC and a CNT wall. The space functions as a capillary to host ion pairs because of diminished effective surface energy.^34^ In the left frame of Figure 3a taken during 3.38 ms, we see a (6,8) crystal bearing a five-ion long shoulder as layer #7. In the next frame, this layer is lost, and the NC has grown in the *y*-direction by one layer, an example of migratory epitaxy (Figure 1c). Given the circular cross-section of CNT, we estimate that layer #7 contained 30 atoms, and the new layer formed at the bottom contained 36 atoms—a reasonable match in the numbers. As shown in Figure 3b, this migration occurred around 2.3052 s during 3.38 ms. Figure 3c,d compares the interlayer distances seen before and after the layer migration. Clearly, the distance between layer #6 and floating layer #7 is 9% longer than the standard 0.282 nm value.

We found that low-frequency mechanical vibration of the CNT container caused migratory epitaxy (Figure 3e, Figure S6). Such a vibration was previously reported to cause a molecule to shuttle inside^16^ and now is found to promote 2-D epitaxy. Simulating the vibration by a quadratic curve (Figure 3f), we estimated the acceleration of the motion of CNT to be extremely small, 4.4 femtometer ms^–2^, and hence the force given to the FI must also be extremely small.

**Figure 4:**
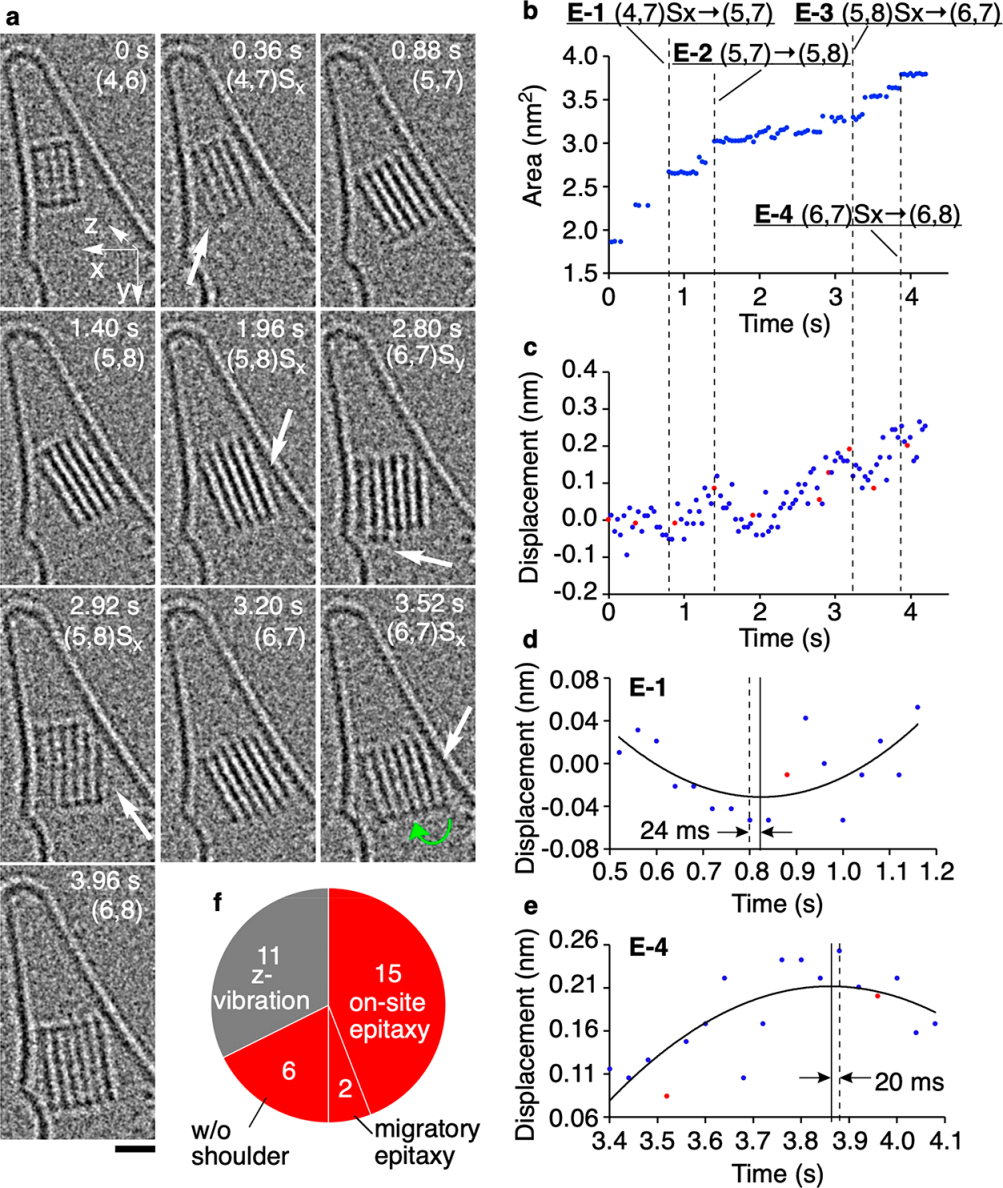
Statistical analyses of crystal growth from a crystal nucleus of NaCl. The NaCl ion pairs were supplied from a hidden reservoir located at the bottom of the CNT.^14^ (a) Representative TEM images in a single event. Time 0 is set arbitrarily. White arrows indicate shoulders in contact with the CNT wall. S_*x*_ and S_*y*_ denote shoulders (white arrows) forming along the *x*- and *y*-directions, respectively. The moiré pattern due to the graphitic lattice was removed by inverse fast Fourier transformation for visibility. Scale bar: 1 nm. (b) Time evolution of the 2-D area of the NaCl NC image. (c) Plot of displacements of CNT wall. Red dots refer to the TEM images shown in (a). (d, e) Expansion figures around 0.8 s corresponding to (4,7)S_*x*_ to (5,7) and around 3.9 s corresponding to (6,7)S_*x*_ to (6,8) growth with a quadratic function fitting of the vibrations. The solid and dotted lines refer to the top of the quadratic curve and the moment of crystal growth, respectively. (f) The ratio of on-site vs migratory epitaxy as studied for 34 events of the growth of NC in a CNT during 118 s with a 40 ms frame rate. The limitation of the TEM observation is that we can identify neither a shoulder nor vibration along the *z*-direction that is shown on the left side of the pie graph.

The nucleation/growth events are strongly correlated to the CNT vibration. Figure 4a shows four examples of the growth during conversion of a (4,6) crystal to (6,8), as analyzed for the size (area) of the NC in Figure 4b. The events E-1 to E-4 are the archetypes of what we observed. E-1 shows on-site epitaxy from (4,7)S_*x*_, (4,7) crystal with a shoulder forming along the *x*-directions, to (5,7), and E-4 shows migratory epitaxy from (6,7)S_*x*_ to (6,8) (migration shown with green arrow). The (5,8)S_*x*_ to (6,7) includes on-site epitaxy and structure reorganization to reduce the surface energy. E-2, a (5,7)-to-(5,8) conversion, does not show any shoulder, possibly because it formed on an invisible face of the crystal. Figure 4b,c illustrates a close correlation between in-plane CNT vibration and growth. As magnified in Figure 4d,e for E-1 and E-4, the growth (dashed line) took place 24 ms before and 20 ms after the CNT vibration reached its maximum amplitude (solid line). Given the femtometer ms^–2^ order acceleration felt by the vibrating CNT (see Figure 3f), we consider that the NC rotation occurred with little energetic cost (i.e., negligible activation energy).

Of the two modes of surface catalyzed epitaxy (Figure 1c), we found that on-site epitaxy overwhelms the migratory epitaxy, as studied for 34 crystal growth events observed during 116 s. The pie graph in Figure 4f summarizes the results. We found a shoulder in 28 cases, and 23 cases of crystal growth triggered by the vibration of the CNT (Figures S7, S8, and S9). Out of the 23 cases, on-site epitaxy took place in 21 cases, and migratory epitaxy only in two cases. By averaging out the increase in the size of the NC over eight observed growth events, we estimate that ∼30 NaCl ion pairs s^–1^ become attached to a NC. This number of the frequency is far smaller than the growth rate of steps in 2-D epitaxy (900 NaCl s^–1^, Figure 2), suggesting that the rate-limiting step of the surface-catalyzed crystal growth is the conversion of a “floating island” to an immobile terrace (cf. Figure 2d).

## Conclusion

A cinematographic study of homoepitaxy on a defect-free crystal facet has revealed an atomistic scenario on how homoepitaxy occurs and how infrequently it occurs. We also found that the heterogeneous nucleation and growth are noticeably accelerated in a vessel vibrating with several Hz and a magnitude of an angstrom. The summary scheme in Figure 1c shows a very diverse time scale of the epitaxial events ranging from the second for the stochastic FI formation to the millisecond for FI migration and explains why crystallization is so erratic.^35,36^ The formation of a mobile FI is a necessary first step of epitaxy, but it occurs only rarely (2 to 10 s) on a defect-free facet. The FI grows larger stochastically and lands on the crystal facet when its interaction with the crystal surface overwhelms the kinetic energy. The landing triggers subsequent 2-D epitaxy. The TEM images and quantitative data (Figures 3 and 4) suggest how the surface of a container can catalyze 2-D epitaxy and crystal growth. A nano space formed between the NC and the CNT wall has a low surface potential, stabilizing FI by the capillary action. When the CNT vibrates, the NC tumbles, the nanospace disappears, the surface potential increases, and the FI forms an immobile terrace, which quickly grows larger (Figure 4). We expect that the surface catalysis found on the nanoscale operates in bulk-scale heterogeneous crystallization because any macroscopic surface undulates on the nanoscale, and low-frequency vibration is ubiquitous. Agitation and attrition have long been known to speed up crystallization—a phenomenon that has attracted people’s curiosity for centuries.^37−39^ We expect that the surface-accelerated epitaxy also operates when a crystal touches another crystal, causing crystal fusion.

## Data Availability

All of the data necessary for evaluating the conclusions of the study are included in the main text or the Supporting Information.

## Supporting Information Available

Additional experimental details, statistical analyses, and representative TEM images (PDF)
Experimental TEM videos (AVI-1, AVI-2, AVI-3)

## Supplementary Material

oc2c01093_si_001.pdf

oc2c01093_si_002.avi

oc2c01093_si_003.avi

oc2c01093_si_004.avi
