# Supplementary material for: Cinematographic Recording of a Metastable Floating Island in Two- and Three-Dimensional Crystal Growth
Source: ACS Cent Sci. 2022 Dec 20;8(12):1704–10. doi: 10.1021/acscentsci.2c01093 (PMC9801501; doi:10.1021/acscentsci.2c01093)
Supplement: Supplementary file 1 — oc2c01093_si_001.pdf [file oc2c01093_si_001.pdf]

# Supporting Information for

Cinematographic Recording of Metastable Floating Island in Two- and Three-Dimensional crystal growth

Masaya Sakakibara[1], Hiroki Nada[2†], Takayuki Nakamuro[1]*, Eiichi Nakamura[1]*

[1] Department of Chemistry, The University of Tokyo, 7-3-1 Hongo, Bunkyo-ku, Tokyo 113-0033, Japan
[2] Environmental Management Research Institute, National Institute of Advanced Industrial Science and Technology (AIST), 16-1 Onogawa, Tsukuba 305-8569, Japan
[†] Current affiliation: Division of Mechanical and Physical Engineering, Faculty of Engineering, Tottori University, 4-101 Koyama-Minami, Tottori 680-8552, Japan.

*Correspondence to: muro@chem.s.u-tokyo.ac.jp (T.N.), nakamura@chem.s.u-tokyo.ac.jp (E.N.)

**This PDF file includes:**

Figure S1 to 9
Captions for Videos S1 to S3

**Other Supplemental Materials for this manuscript include the following:**

Videos S1 to S3

**Table of Contents**

**1. Methods** S2

**2. Supplemental figures** S5

**3. Representative TEM images** S15

**4. Legends for Supplemental Video: Videos S1-S3** S16

## Methods

### General

Chemicals were purchased from Tokyo Kasei Co., Aldrich Inc., and other commercial suppliers and used after appropriate purification before use. A black powder of aggregate of conical carbon nanotubes (CNT) was purchased from NEC Co. (Carbon Nanohorns, Lot No. 181-3-2; assay: 90%) and used as purchased. Distilled water was further purified with Millipore Milli-Q (Milli-Q Reference, Merck). TEM grid precoated with a lacy microgrid (NS-C15, pore size 1.5–8 μm) was purchased from Okenshoji Co., Ltd.).

### TEM instrumentation

Atomic-resolution transmission electron microscopic (TEM) observation was carried out on a JEOL JEM-ARM200F instrument equipped with an aberration corrector (point resolution: 0.10 nm) at 298 K and at an acceleration voltage of $E$ = 80 kV, under $1 \times 10^{-5}$ Pa in the specimen column. We used a spherical aberration ($C_s$) value of between 1 and 3 μm, and an electron dose rate (EDR; the number of electrons per second per nm$^2$) of $4.0 \times 10^5$ – $4.6 \times 10^6$ $e^-$ nm$^{-2}$ s$^{-1}$ at × 800,000–2,000,000 magnification. For × 2,000,000 magnification observation, a series of images were continuously recorded at a frame rate of 40 ms frame$^{-1}$ on a complementary metal-oxide-semiconductor camera (Gatan OneView, In situ mode, 4096 × 4096 pixels) operating on a binning 2 mode (output image size: 2048 × 2048 pixels, pixel resolution 0.01 nm at × 2,000,000). For × 800,000 magnification observation, a series of images were continuously recorded at a frame rate of 1.69 ms frame$^{-1}$ on a direct electron detection camera (Gatan K3, In situ mode, Counted mode, 5760 × 4092 pixels) operating on a binning 1 mode and 1/2 ROI (output image size: 2048 × 2048 pixels, pixel resolution 0.005 nm at × 800,000). All images were automatically processed on a Gatan DigitalMicrograph software. To record the atomic-resolution videos of the specimens, we first surveyed the whole CNT aggregates on the grid at × 100,000 magnification to find conical CNTs suitable for careful analysis. To analyze them in depth, we increased the magnification to × 800,000–2,000,000 and started the video recording. We adjusted the defocus value during image collection. The images were recorded at under-focus conditions (defocus value: –10 nm), a typical value that offers the best compromise between image contrast and richness of structural information. The images were recorded in a .dm4 format using the Gatan DigitalMicrograph software.

### TEM image processing

The images taken in a .dm4 format were transformed into 8-bit or 32-bit .tiff format file by Gatan DigitalMicrograph and Fiji software.[1]

The data sets recorded on OneView were aligned to minimize specimen drift and rotation by checking the cross-correlation of each image and filtered by a bandpass filter (filtering structures smaller than 5 pixels and larger than 40 pixels, tolerance of direction: 5%). When the moiré pattern of graphitic lattice interferes the NaCl cluster/crystal images, we remove it with the fast Fourier transform software implemented in Fiji software.[2] We herein define 'Binning = X × Y × T' as combining a cluster of X × Y × T (X-axis, Y-axis, and time-direction), and pixel binning (4 × 4 ×

2 pixels into 1 pixel, pixel resolution 0.020 nm, frame rate 3.38 ms $frame^{-1}$) and lowpass filter (filtering structures smaller than 3 pixels, tolerance of direction: 5%) were applied to the data sets recorded on K3 for smoothing the images. Optimization of the K3 image processing procedure is shown in Figure S1. Linear adjustment of brightness and contrast was applied as required for analyses. Gaussian filter ($\sigma_x = \sigma_y$ = 2.5 pixels, $\sigma_z$ = 0.7 frame) was also applied on processed images for improving visibility for presentation (Figure S1).

**TEM image simulation**

Each experimental TEM image was analyzed through comparison with the simulated images of putative models of NaCl crystals in a CNT (NaCl@CNT) seen from various directions. The image simulation was performed by using a multi-slice procedure implemented in a Bionet elbis software.[3] We used the experimental TEM conditions including total electron dose as the parameters for the simulation. The model of NaCl@CNT for the simulation was generated on a MaterialsStudio® software by fitting a NaCl crystal in a chemical model of CNT whose atomic coordinate was generated to reproduce the outline of the TEM image (Figure S2). For NaCl we used the effective ionic radii of $Na^+$ and $Cl^-$.[4,5]

**Encapsulation of NaCl in amino-CNT (NaCl@amino-CNT)**

Amino-CNT (3.0 mg)[6] was dispersed at 298 K in saturated aqueous NaCl solution (99.5% pure, 1 mL using MilliQ water). After stirring for 24 hours, the reaction mixture was filtered through a PTFE membrane filter (ADVANTEC, pore size: 0.2 μm) and a black filter cake was washed with methanol (1 mL × 3). The black powder was placed in vacuo (60 Pa) for 30 minutes to obtain NaCl@amino-CNT (3.1 mg) which was analyzed by single-molecule atomic-resolution time-resolved electron microscopy (SMART-EM).

The black powder of NaCl@amino-CNT forming a large agglomerate was wetted by methanol (2 mL/mg), and gently ground for 3 minutes in an agate mortar so as to break the agglomerate into individual CNT aggregates for TEM analysis. The dispersion was filtered through Kiriyama 5A filter (pore sizes: 7 μm), and the filtrate (10 μL) was drop casted onto a TEM microgrid placed on a paper that absorbed excess methanol. The resulting TEM grid was placed in vacuo (60 Pa) at 298 K for 1 hour.

**Measurement of mechanical vibration of CNT**

The CNTs underwent stochastic mechanical vibration during TEM observation, for reasons yet to be probed. Mechanical vibration of CNT was evaluated by measuring the position of graphitic wall of CNT (2.0 nm from tip of the CNT) shown in Figure S4. To avoid the influence of Gaussian filtering on the measured values, measurements were conducted on the original images, and the filtered, highly visible images are shown for presentation purposes only (cf., Figure 3a). Due to thermal drift of the specimen from right to left, the displacement gradually decreased.

**Correlation between crystal growths and CNT mechanical vibration**

In this study, mechanical vibrations with observed displacements larger than 0.1 nm were analyzed. The maximum displacement observed was 0.2 nm, which indicates that two-thirds (i.e., 240°/360°) of the whole vibration was analyzed (Figure S5). As shown in Figure 4f, the present analysis shows that 67.6 % of the crystal

growths are correlated with the vibration. This result is consistent with the hypothesis that crystal growths are induced by the mechanical vibration of CNT.

TEM images shown in Figure 4 are taken from $8^{th}$ event of 9-repetitive crystallization, and not published in our previous report.[7] In the previous paper, we demonstrated the crystallization of $1^{st}$ event. Note that manual movement during 3.0-3.3 s in vibrational plot shown in Figure 4c was corrected by subtracting the movement, and the vibrational correlation of E-3 was neglected to avoid any incorrect interpretation.

**Computational study on the structure and potential energy for NaCl surface clusters**

The potential energy difference, $\Delta U$, which was the difference in the potential energy, $U$, between each $r$ (> 0) and $r = 0$ (Figure 2g), the distance between the top NaCl layer of the crystal and the cluster in the $x$ direction, $d$ (Figure 2h), and the Na–Cl bond length in the cluster (Figure 2i, j) in the manuscript were obtained with Monte Carlo (MC) simulations in the following way. First, a MC simulation was performed for a system in which a 16-atomic square NaCl cluster was placed at a certain position ($r = 0$) on one of the two $y$–$z$ surfaces ($x \geq 0$) of the cubic crystal (Figure 2f). During the MC simulation, the positions of ions in the crystal were fixed at their equilibrium positions at 298 K. The maximum displacement in the trial movement of each ion in the cluster for each of $x$, $y$, and $z$ directions was set to 0.01 nm. $U$, $d$, and the Na–Cl bond length in the cluster for $r = 0$ were estimated using $5 \times 10^3$ configurations, which were obtained at every $4 \times 10^2$ trial movements of ions after equilibration of $2 \times 10^6$ trial movements of ions. The final configuration of the cluster in this MC simulation for $r = 0$ was used as the initial configuration of the cluster in an MC simulation for each $r$ (> 0) along the $\langle 100 \rangle$ and $\langle 110 \rangle$ directions.

The MC simulations for $r > 0$ were also performed using the crystal in which positions of ions was fixed at their equilibrium positions at 298 K. During the MC simulations for $r > 0$, ions in the cluster were allowed to move only in the $x$ direction with the maximum displacement of 0.03 nm. $U$, $d$, and the Na–Cl bond length in the cluster for $r > 0$ were estimated using $1 \times 10^3$ configurations, which were obtained at every $1.6 \times 10^3$ trial movements of ions after equilibration of $4.8 \times 10^6$ trial movements of ions.

All MC simulations were performed using a standard Metropolis sampling method at a constant temperature of 298 K. The interaction acting on each ion was estimated with a model proposed by Joung and Cheatham.[8] In this model, the interaction between a pair of ions is represented as the Coulomb interaction plus the Lennard-Jones interaction. Calculation of the interaction was performed for all pairs of ions in the system.

## Supplementary Figures

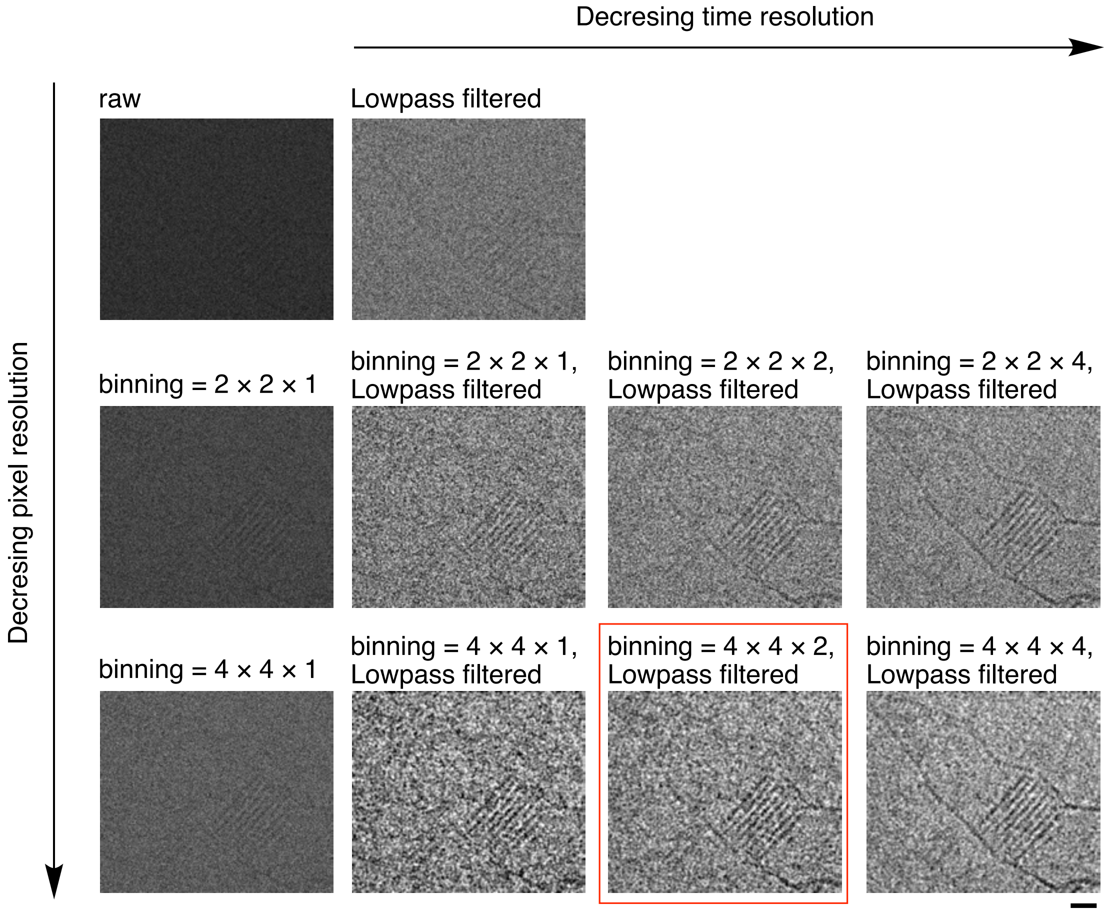


**Figure S1.** Optimization of K3 image processing. 'Binning = X × Y × T' means combining a cluster of X × Y × T (X-axis, Y-axis, and time-direction) pixels into 1 pixel. EDR = $4.6 \times 10^6$ $e^-$ $nm^{-2}$ $s^{-1}$, Magnification × 800,000, Scale bar: 1 nm.

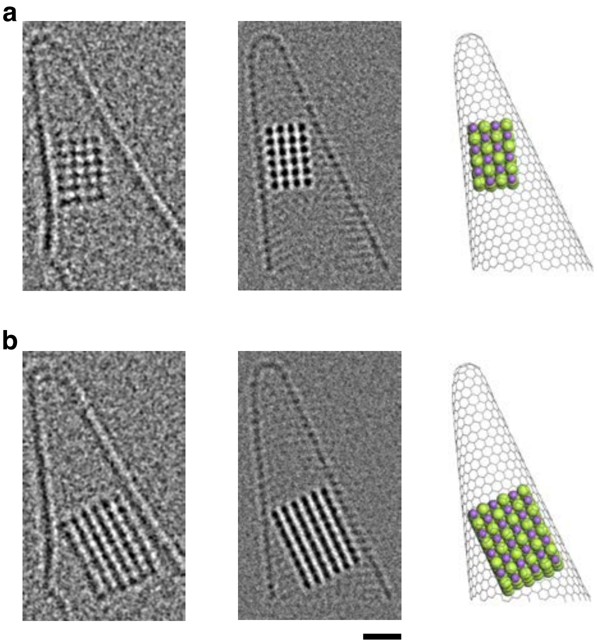


**Figure S2.** TEM images, TEM simulations and structural models for (a) (4,6) and (b) (6,8) nanocrystals are shown from left to right. Scale bar: 1 nm. The assignment on which of $Na^{+}$ and $Cl^{-}$ occupies the corners of crystals are arbitrary because the TEM image lacks this information.

## Sequential images of NaCl growth taken by K3-IS

Representative 40 frames of lateral growth of NaCl in frame rate of 3.38 ms frame$^{-1}$ without Gaussian filtering are shown in Figure S3. Sequential 6 frames of migratory epitaxy of NaCl in frame rate of 3.38 ms frame$^{-1}$ without Gaussian filtering are also shown in Figure S4.

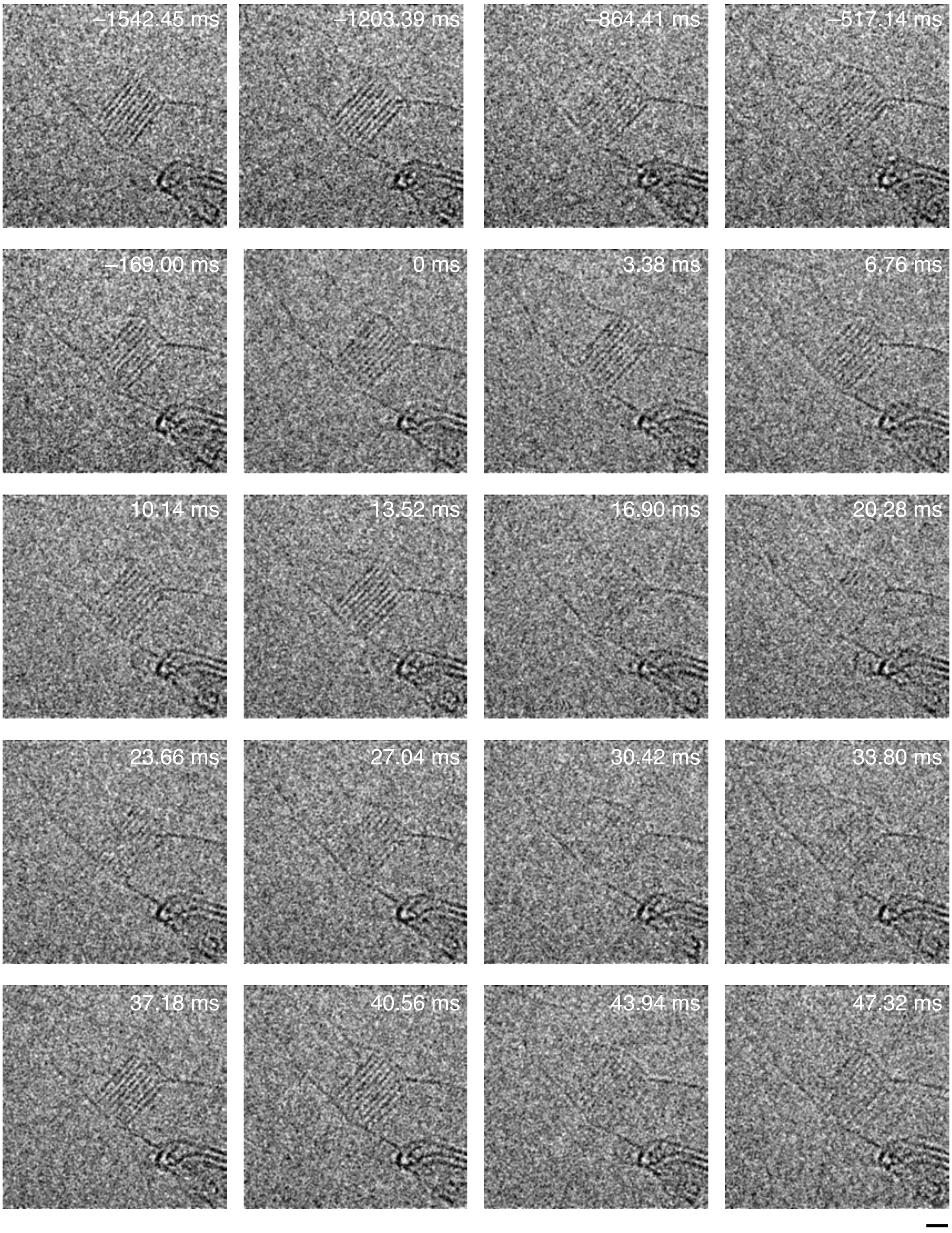


**Figure S3-1.** K3-IS images of the NaCl lateral growth in Figure 2. Acceleration voltage 80 kV and EDR of 4.6 × $10^6$ $e^-$ $nm^{-2}$ $s^{-1}$. Scale bar: 1 nm.

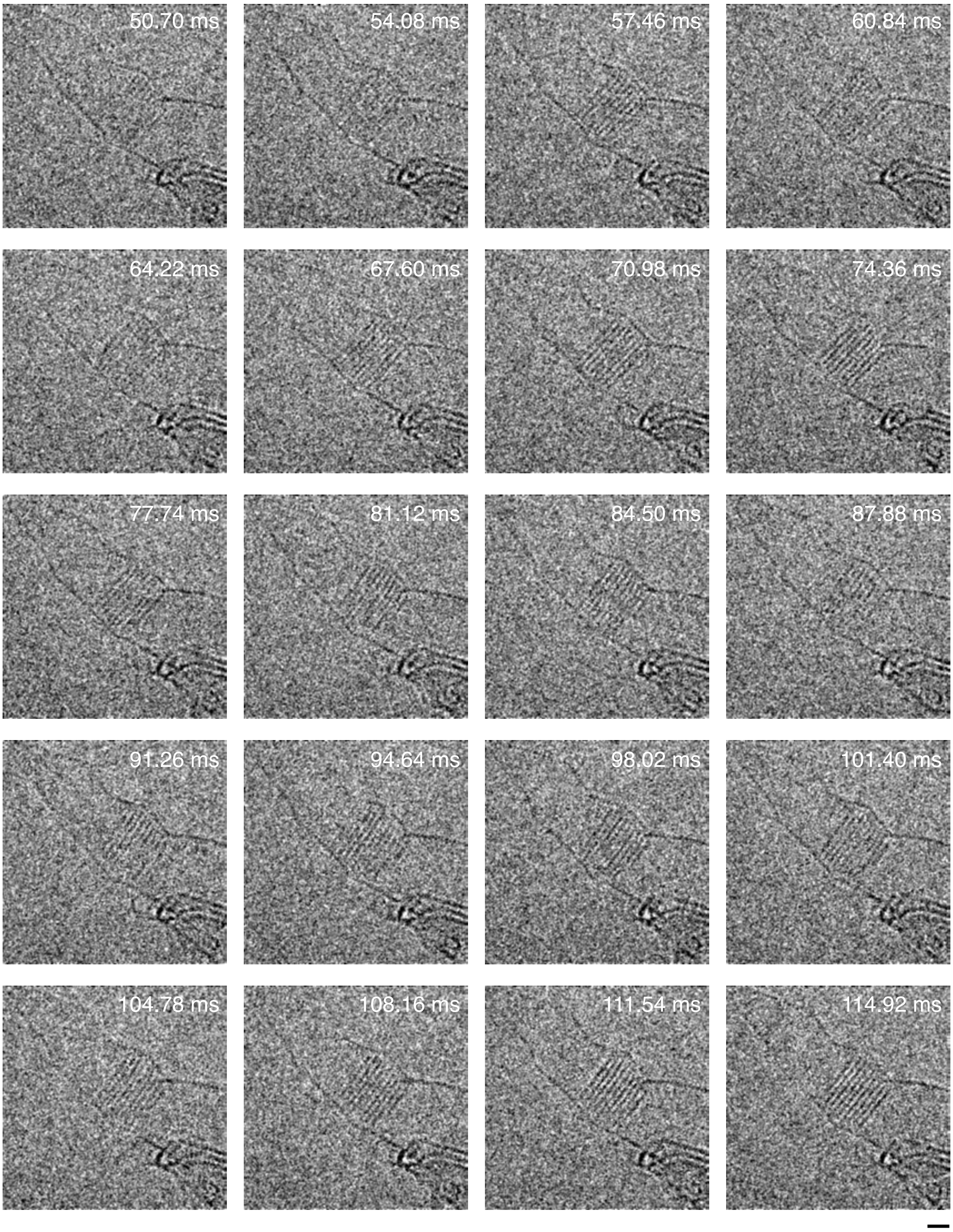


**Figure S3-2.** K3-IS images of the NaCl lateral growth in Figure 2. Acceleration voltage 80 kV and EDR of $4.6 \times 10^6$ $e^-$ $nm^{-2}$ $s^{-1}$. Scale bar: 1 nm.

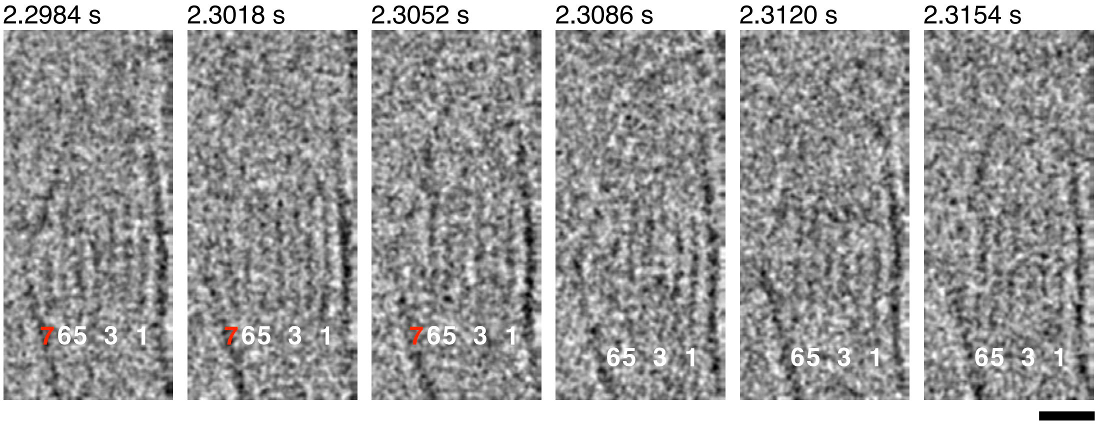


**Figure S4.** K3-IS images of the NaCl migratory growth in Figure 3. Acceleration voltage 80 kV and EDR of $2.2 \times 10^6$ $e^-$ $nm^{-2}$ $s^{-1}$. Scale bar: 1 nm.

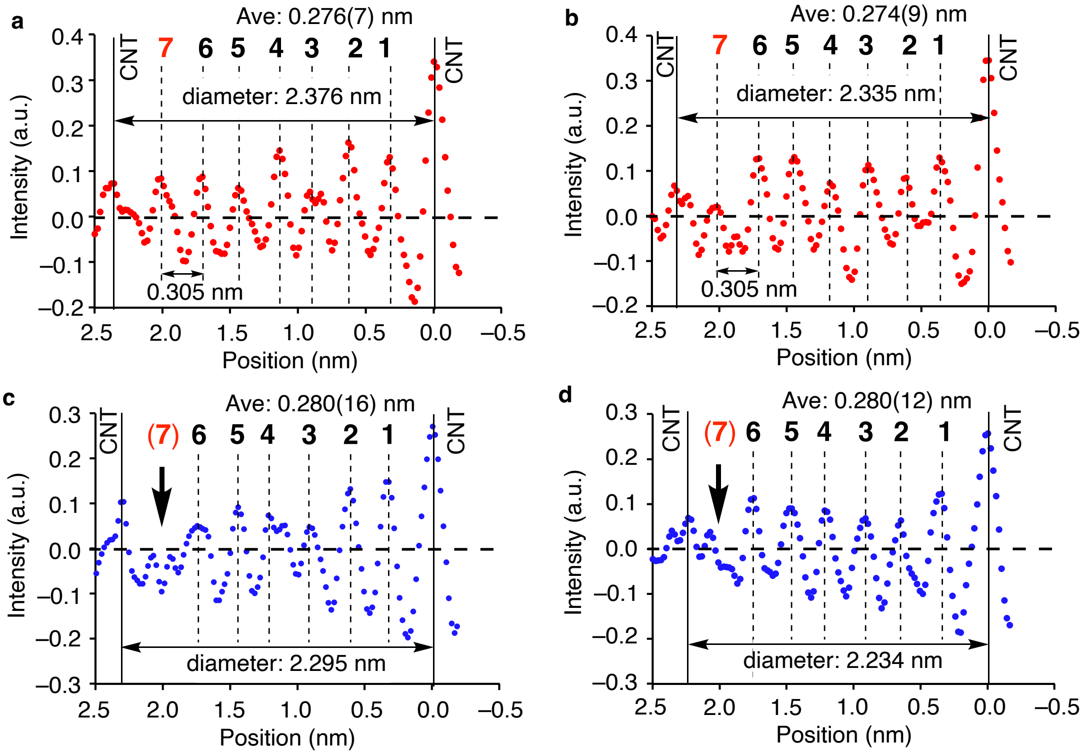


**Figure S5**. Plot profiles taken from Figure 3b. (a)-(d) Interlayer distance measurement before (2.3018 and 2.3052 s) and after (2.3086 and 2.3120 s) migratory epitaxy.

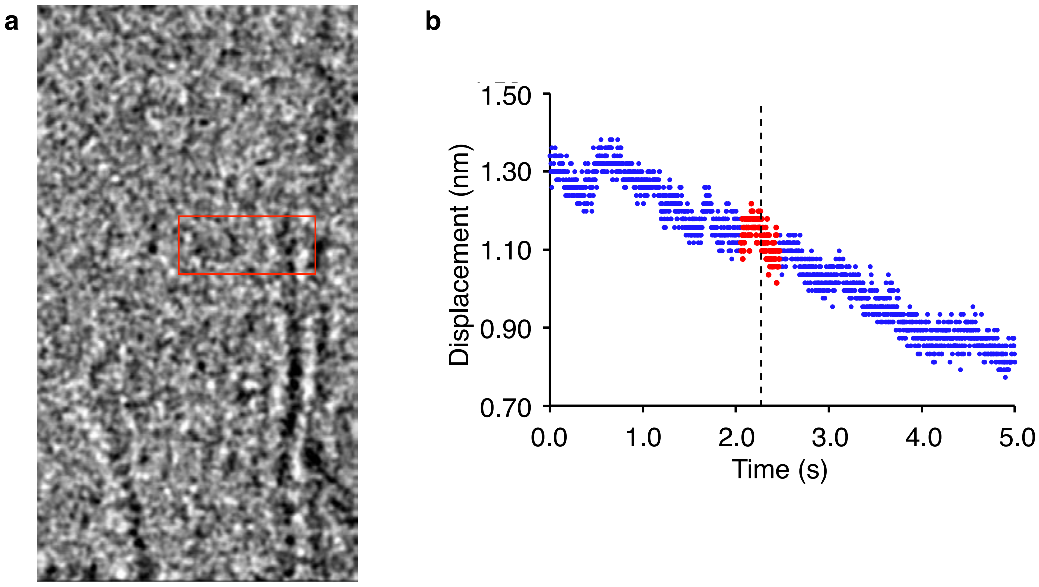


**Figure S6**. Analysis of CNT vibration. (a) Representative TEM image with a red rectangle indicating the measured position. (b) Vibrational plot shown in Figure 3e. Displacement was measured on the red rectangle from left to right Due to thermal drift of specimen from right to left, the displacement gradually decreased.

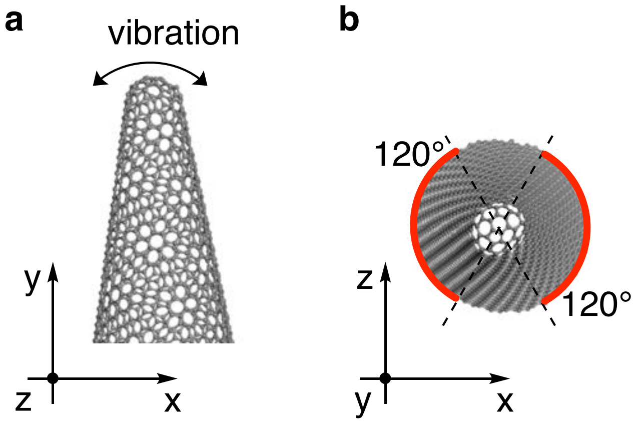


**Figure S7.** Schematic diagrams of the analysis of CNT vibration. (a) Side view of vibrating CNT. (b) Top view of CNT. Measured range of vibration angle are highlighted in red (240°/360°).

**Details in interlayer distance measurements on surface catalyzed epitaxy**

Interlayer distances on surface clusters transiently formed in surface-catalyzed epitaxy were statistically analyzed (Figure S8). The result clearly shows the correlation between *d* and the mobility of the surface clusters (more mobile cluster exhibits larger *d* value). 27 individual images are also shown in Figure S9.

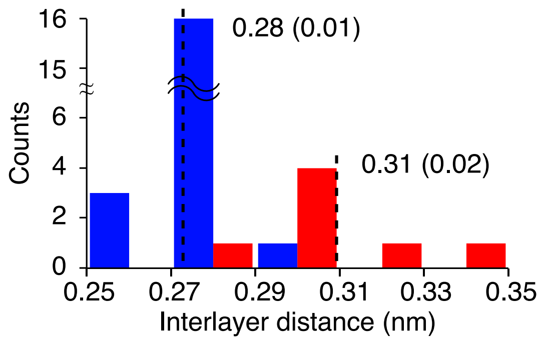


**Figure S8.** Statistical analysis of *d* detected in surface-catalyzed epitaxy. $N = 27$. Blue: On-site epitaxy, Red: Migratory epitaxy. Dashed lines indicate the average *d* value.

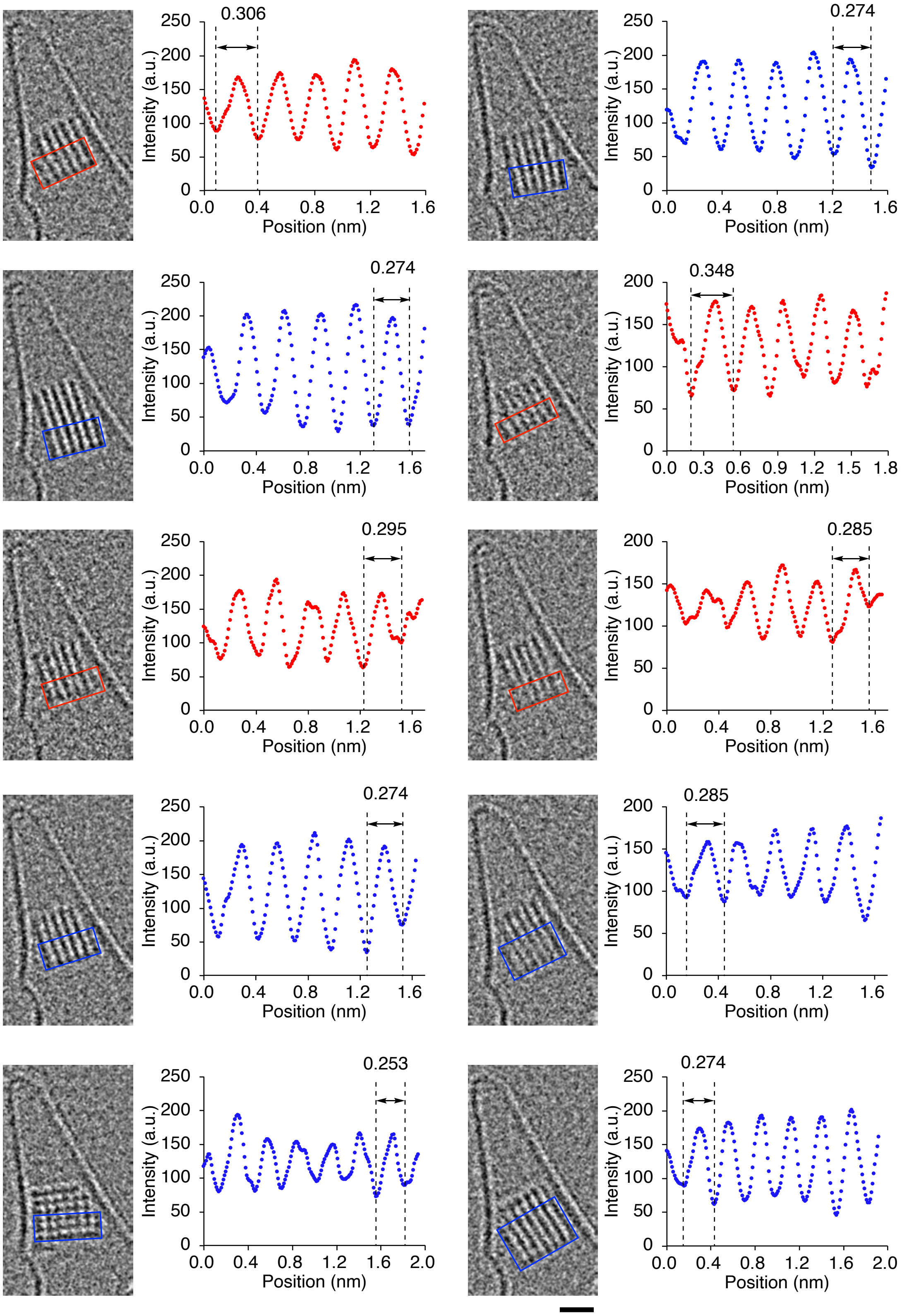


**Figure S9-1.** Detailed results of interlayer distance measurement in Figure S8. EDR is $4.0 \times 10^5$ $e^-$ $nm^{-2}$ $s^{-1}$. Scale bar: 1 nm. Rectangles represent measured areas. Blue: On-site epitaxy, Red: Migratory epitaxy.

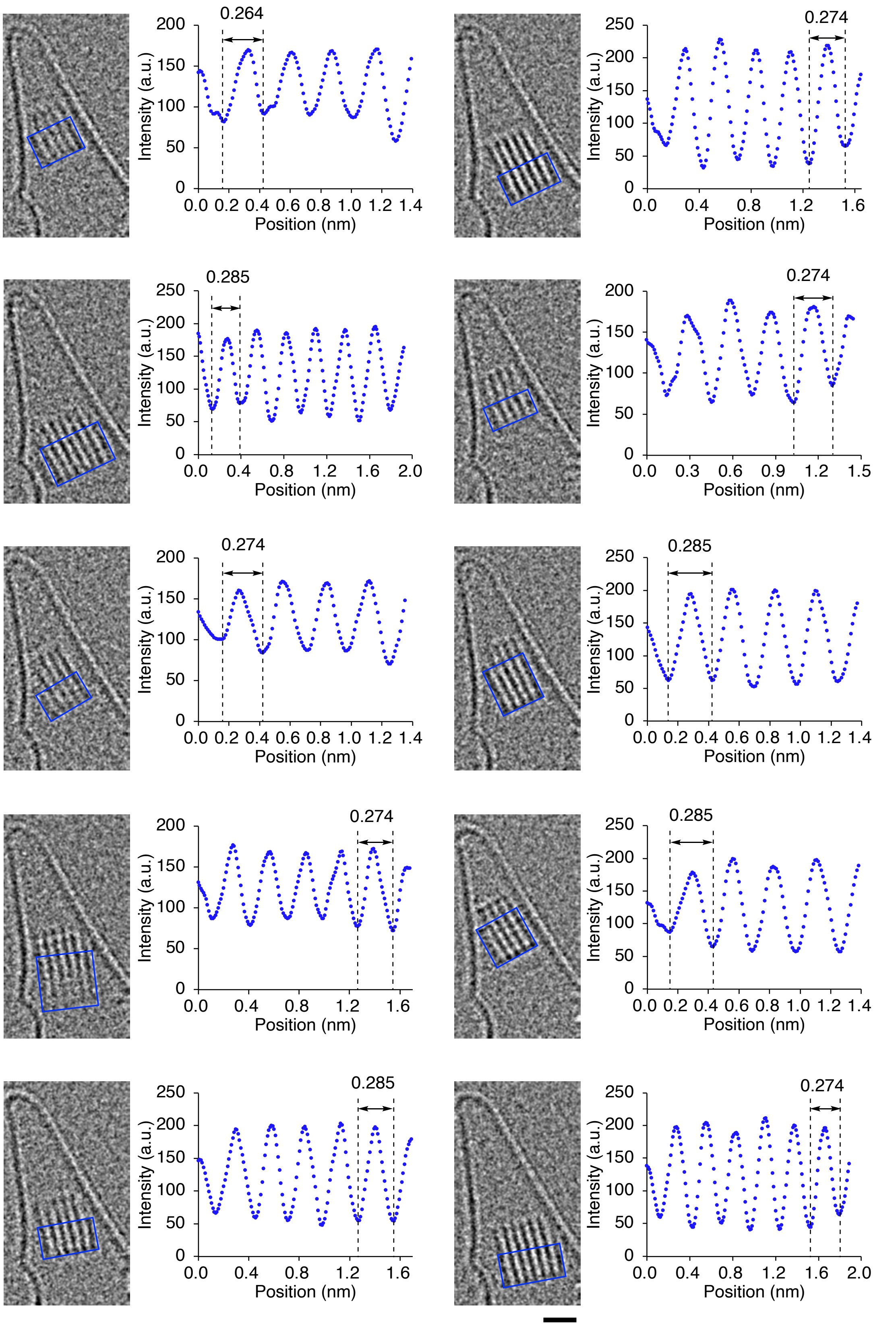


**Figure S9-2.** Detailed results of interlayer distance measurement in Figure S8. EDR is $4.0 \times 10^5$ $e^-$ $nm^{-2}$ $s^{-1}$. Scale bar: 1 nm. Rectangles represent measured areas. Blue: On-site epitaxy, Red: Migratory epitaxy.

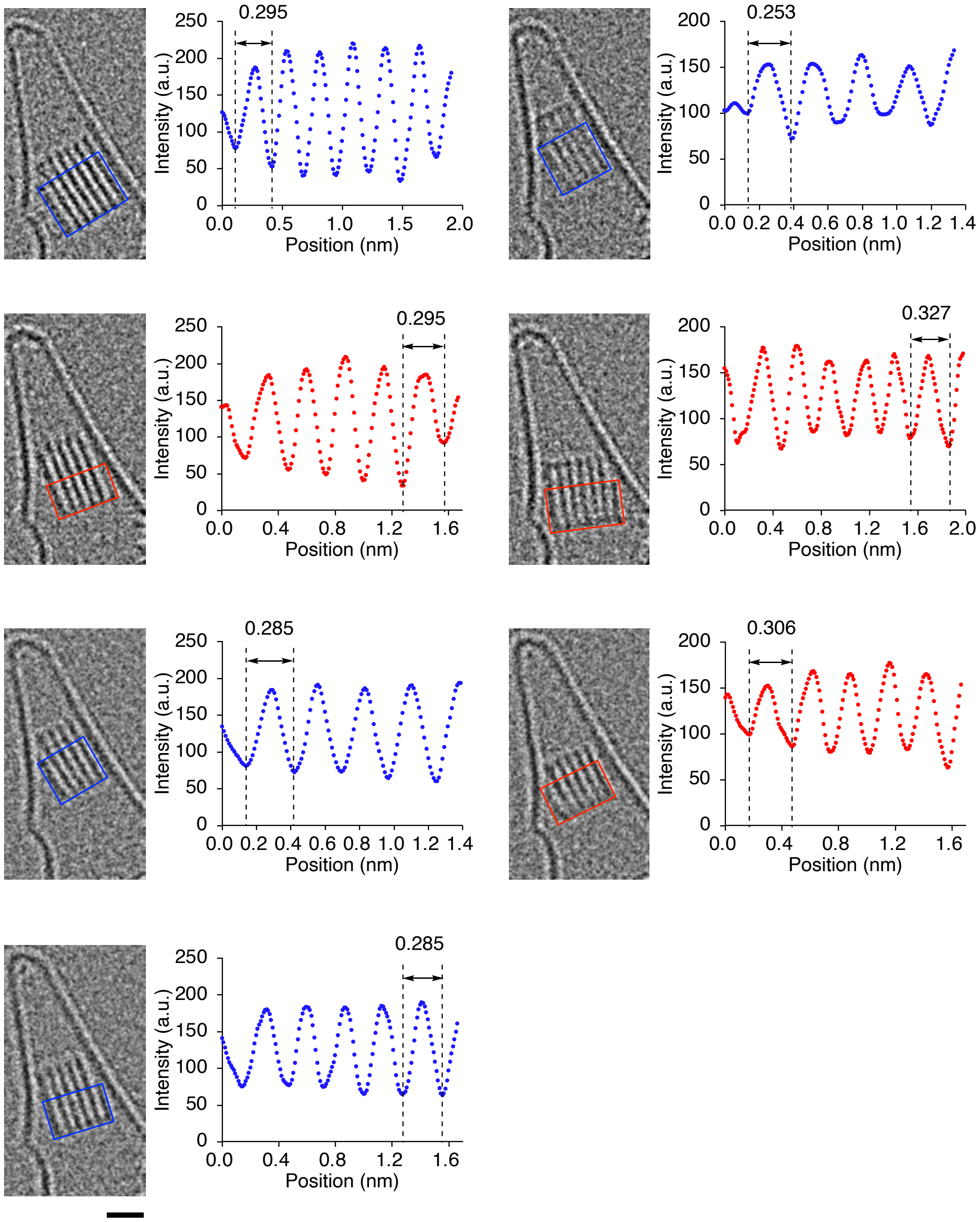


**Figure S9-3.** Detailed results of interlayer distance measurement in Figure S8. EDR is $4.0 \times 10^5$ $e^-$ $nm^{-2}$ $s^{-1}$. Scale bar: 1 nm. Rectangles represent measured areas. Blue: On-site epitaxy, Red: Migratory epitaxy.

**Legends for Supplemental Videos**

**Video S1. Formation of FI and on-site epitaxial growth on a NaCl NC.** This is the video of surface migration and subsequent lateral growth of NaCl after processing. The experimental conditions were an acceleration voltage of 80 kV, electron dose rate of $4.6 \times 10^6$ $e^-$ $nm^{-2}$ $s^{-1}$, and exposure time of 3.38 milliseconds for each frame. The playback speed of the video is one-tenth of the original video recording.

**Video S2. Millisecond-scale TEM video of the on-site epitaxy without Gaussian filtering.** This is the video same with Video S1 before Gaussian filtering. All analyses shown in the main text were conducted on this video. The experimental conditions were an acceleration voltage of 80 kV, electron dose rate of $4.6 \times 10^6$ $e^-$ $nm^{-2}$ $s^{-1}$, and exposure time of 3.38 milliseconds for each frame. The playback speed of the video is one-tenth of the original video recording.

**Video S3. Migratory epitaxy of a NaCl NC.** This is the video of migratory growth of a NaCl NC after processing. The experimental conditions were an acceleration voltage of 80 kV, electron dose rate of $2.2 \times 10^6$ $e^-$ $nm^{-2}$ $s^{-1}$, and exposure time of 3.38 milliseconds for each frame. The playback speed of the video is one-tenth of the original video recording.

## References


1. Schindelin, J.; Arganda-Carreras, I.; Frise, E.; Kaynig, V.; Longair, M.; Pietzsch, T.; Preibisch, S.; Rueden, C.; Saalfeld, S.; Schmid, B.; Tinevez, J.-Y.; White, D. J.; Hartenstein, V.; Eliceiri, K.; Tomancak, P.; Cardona, A. Fiji: an open-source platform for biological-image analysis. *Nat. Methods* **2012**, *9*, 676–682.
2. Kim, Y.-M.; Jeong, J.-M.; Kim, J.-G.; Kim, Y.-J.; Lim, Y. S. Image processing of atomic resolution transmission electron microscope images. *J. Korean Phys. Soc.* **2006**, *48*, 250–255.
3. Hosokawa, F.; Shinkawa, T.; Arai, Y.; Sannomiya, T. Benchmark test of accelerated multi-slice simulation by GPGPU. *Ultramicroscopy* **2015**, *158*, 56–64.
4. Shannon, R. D.; Prewitt, C. T. Effective ionic radii in oxides and fluorides. *Acta Cryst*. **1969**, *B25*, 925–946.
5. Shannon, R. D. Revised effective ionic radii and systematic studies of interatomic distances in halides and chalcogenides. *Acta Cryst*. **1976**, *A32*, 751–767.
6. Hanayama, H.; Yamada, J.; Harano, K.; Nakamura, E. Cyclodextrins as surfactants for solubilization and purification of carbon nanohorn aggregates, *Chem. Asian J.* **2020**, *15*, 1549–1552.
7. Nakamuro, T.; Sakakibara, M.; Nada, H.; Harano, K.; Nakamura, E. Capturing the moment of emergence of crystal nucleus from disorder. *J. Am. Chem. Soc.* **2021**, *143*, 1763–1767.
8. Joung, I. S.; Cheatham, T. E. Determination of alkali and halide monovalent ion parameters for use in explicitly solvated biomolecular simulations. *J. Phys. Chem. B* **2008**, *112*, 9020–9041.
